# The significance of widely split P waves: a case report

**DOI:** 10.1186/s13256-022-03432-5

**Published:** 2022-05-21

**Authors:** Marianne C. Chen, Jay B. Brodsky

**Affiliations:** Department of Anesthesiology, Perioperative and Pain Medicine, Stanford University School of Medicine, Stanford, CA 94305 USA

**Keywords:** P wave morphology on electrocardiogram, Split P waves, Mobitz type II arrhythmia, Case report

## Abstract

**Background:**

P wave morphology on electrocardiogram is often overlooked but indicates abnormal cardiac conduction from various etiologies. Split P waves on electrocardiogram have been reported previously but not in a perioperative setting.

**Case presentation:**

A 69-year-old Caucasian male patient with widely split P waves on his preoperative electrocardiogram was scheduled for a reimplantation right total hip replacement under a combined spinal–general anesthetic technique. The patient was evaluated prior to surgery by a cardiologist and the preoperative anesthesia clinic without any comment on the abnormal P wave morphology on electrocardiogram. The patient was cleared to proceed with anesthesia and surgery. Following induction of general anesthesia, his cardiac rhythm changed to a Mobitz type II pattern. The surgical procedure was cancelled, and a permanent cardiac pacemaker was inserted.

**Conclusions:**

Anesthesiologists should be aware that the presence of widely split P waves on electrocardiogram indicates the presence of atrial conduction abnormalities, likely from an ischemic or infiltrative process that can lead to more serious cardiac arrhythmias. P wave morphology should be observed and noted during the perioperative period for all patients.

## Background

Abnormal P wave morphology on an electrocardiogram (ECG) may be overlooked during an anesthetic procedure. We wish to draw attention to the potential danger associated with the presence of widely split P waves on ECG, indicating atrial conduction abnormalities likely from structural heart disease. A few case reports in cardiology literature have described the occurrence of split P waves, but it has not been described previously in a patient undergoing a surgical procedure with anesthesia [1, 2]. The CARE guidelines (CAse REports) were used in the preparation and writing of this case report. Written HIPAA authorization was obtained from the patient.

## Case description

**Fig. 1:**
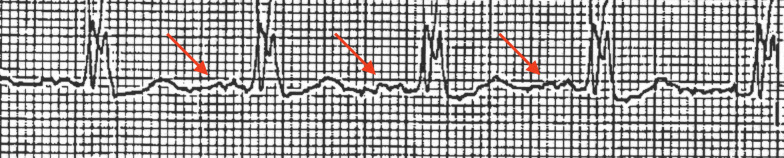
Preoperative electrocardiogram showing split P waves, highlighted by arrows

A 69-year-old Caucasian man was admitted to the hospital for reimplantation right total hip replacement. He had undergone a resection right hip arthroplasty 5 months prior to this admission for a hardware infection from a previous right hip fracture repair. His past medical history included coronary artery disease s/p right coronary artery bare metal stent 12 years ago, congestive heart failure, diabetes mellitus, hypertension, and depression. Medications included carvedilol 3.125 mg two times a day, furosemide 40 mg two times a day, glargine insulin 13 units every bedtime, escitalopram 20 mg once daily, pravastatin 40 mg daily, and omeprazole 20 mg daily. Preoperative laboratory studies were all within normal limits. A transthoracic echocardiogram was performed 2 weeks prior to surgery, which showed moderate left ventricular hypertrophy with low–normal left ventricular systolic function without any valvular abnormalities. His preoperative ECG was read as sinus rhythm with a rate of 74 beats per minute (bpm), left-bundle branch block, and probable left atrial abnormalities. On closer examination of his ECG, he was noted to have split P waves, most distinct in leads I and AVL, appearing like two distinct P waves (Fig. 1). PR interval was 204 milliseconds, and P wave duration about 150 milliseconds. He had been evaluated by his cardiologist, who cleared him for surgery and did not require any further testing. There was no comment about the split P waves on ECG by the cardiologist. The patient was also evaluated by the pre-anesthesia clinic, who deemed him appropriate to proceed with anesthesia and surgery. The patient requested spinal anesthesia, but given that the reimplantation surgery was scheduled to take up to 3–4 hours, we elected to proceed with a combination spinal and general anesthesia. The decision to proceed with spinal anesthesia was to be able to use intrathecal morphine as the primary analgesic and thus minimize the need for intravenous opioids. The local anesthetic with spinal anesthesia would also then allow for a lighter general anesthetic, which would still be needed given the potential length of the surgery.

**Fig. 2:**
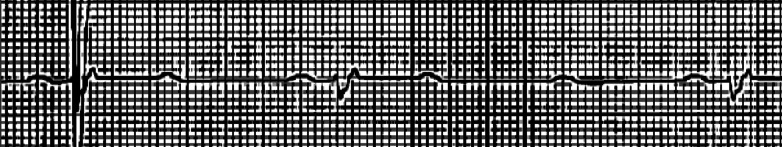
Intraoperative electrocardiogram: second degree atrioventricular block, Mobitz type II

On the day of surgery, the patient was taken to the operating room and standard ASA monitors were applied. His mean arterial pressure (MAP) was 66 mmHg, and his heart rate was 50 bpm. The patient’s bradycardia was thought to likely be due to his beta-blocker medication. Prior to surgery, he denied any recent symptoms including chest pain, palpitations, or syncopal events. The patient’s previous medical records showed that his heart rate was usually in the 50–70 bpm range. Given the patient’s recent cardiac evaluations and the anesthesiologist being unaware of the significance of split P waves, it was assumed that the patient’s bradycardia would be responsive to medications if it worsened with anesthesia. A subarachnoid block was performed at the L3–4 interspace, and 1.3 ml 0.75% bupivacaine with 200 mcg preservative-free morphine was injected. The patient was placed supine, and a T10 sensory level was demonstrated. His MAP was 55 mmHg with a heart rate of 45 bpm. The display monitor demonstrated sinus bradycardia, left-bundle branch block, and widely split-P waves. He was given two doses of glycopyrrolate without increase in heart rate, although his MAP increased with an IV fluid bolus. Given improvement in hemodynamics, a decision was made to proceed with the operation and general anesthesia. Following preoxygenation, anesthesia was induced with fentanyl and propofol, and rocuronium was given for muscle relaxation. The patient’s trachea was intubated without difficulty, and he was mechanically ventilated with oxygen and sevoflurane. Within minutes after anesthetic induction, his heart rate dropped to 30–40 bpm, while his MAP ranged between 60 and 68 mmHg. He was given two doses of atropine and then ephedrine without an increase in heart rate. At this time, a Mobitz type II heart block first appeared on the display monitor. A 12-lead ECG obtained in the operating room confirmed this diagnosis (Fig. 2). The risk of proceeding was discussed with the surgeon, and a decision was made to cancel the surgery. Cardiology was consulted in the operating room, and given stable blood pressures, the cardiologist recommended proceeding with anesthesia emergence and extubation with plans to further evaluate the patient in the recovery room. They recommended against transcutaneous or transvenous pacing at this time. Muscle relaxation was reversed, the sevoflurane was discontinued, and the patient’s trachea was extubated. His MAP remained at 60–70 mmHg with a heart rate of 35–45 bpm.

In the post-anesthesia care unit, he was seen by a cardiologist, who confirmed the Mobitz type II heart block and discussed the need for pacemaker implantation with the patient and his wife. The patient was then immediately taken to the electrophysiology laboratory, where a permanent dual-chamber pacemaker was implanted under conscious sedation. He tolerated this procedure without problems. During the remainder of his hospitalization, he required 100% pacing, atrial-sensed ventricularly paced. The cardiologist noted that the cause of heart block was likely due to an ischemic versus infiltrative process as suggested by his history of right coronary artery myocardial ischemia requiring stenting. He was discharged home 2 days later. He returned to the hospital and underwent a reimplantation right total hip replacement 1 week later under a combined spinal plus general anesthetic technique without any perioperative complications.

## Discussion and conclusions

Although the occurrence of widely split P waves was described over 75 years ago, it is a relatively rare occurrence [3]. Our review of the medical literature revealed only two previous clinical reports of this phenomenon, both associated with significant bradycardia [1, 2]. Soejima *et al.* reported a case of marked intraatrial conduction delay with widely split P waves in a patient with 10-year history of syncope [1]. Ariyarajah *et al.* described a patient with transient, widely split P waves in the presence of intraatrial block [2].

The P wave on an ECG captures the cardiac electrical depolarization at the sinoatrial node directed from right to left atrium. Intra- or interatrial conduction disturbances can increase P wave duration, sometimes creating a notched or biphasic wave, but usually not splitting the P waves [4]. Split P waves are believed to be due to atriopathy, or atrial myocardial lesions resulting in abnormal electrophysiologic conduction. These lesions may develop from ischemia or an infiltrative process such as fibrosis [3]. The right and left atria are independent anatomic compartments separated by a fibrous septal wall; however, they are coupled electrically during sinus rhythm by Bachmann’s bundle. A study of P wave mapping demonstrated that endocardial pacing at the lower-right medial atrium or the high, middle, or lower-left atrium generates four, clearly distinct P wave patterns [5]. This would suggest that a conduction disturbance from the sinus node to the right or left atrium could produce a split P wave. More recently, Sternick *et al.* reported electrophysiology imaging of a patient with split P waves and bradycardia requiring pacemaker implantation. They showed that intraatrial conduction delay must be extreme in cases of split P waves for the right and left atrium to have separate activation and contraction yet still maintain electrical coupling [6]. The cause of split P waves in the Sternick case was related to abnormal glycogen deposit in the myocytes, while the cause of split P waves in the Soejima case was probably related to fibrosis. Interatrial conduction blocks are usually associated with left atrial enlargement and atrial tachyarrhythmias such as atrial fibrillation, which was not found in our patient or the other patients described in the previous case reports.

Although our patient had split P waves on his preoperative and intraoperative ECGs, we were unaware of the potential significance of this finding and therefore proceeded with the planned anesthetic. He developed significant bradycardia after placement of the subarachnoid block. It is well known that spinal anesthesia can cause bradycardia. Since only a T10 sensory level was achieved in the absence of significant hypotension, it is unlikely that the cardioaccelerator fibers were blocked [7]. The patient was taking medications including escitalopram and carvedilol, which could also contribute to bradycardia and arrhythmias, but the patient had normal heart rate in the 70s and no previous history of arrhythmias prior to his surgery despite taking those medications for many years. The bradycardia was unresponsive to atropine, glycopyrrolate, and ephedrine, but since he was otherwise stable and his baseline heart rate was in the 50s, we made the decision to proceed with general anesthesia. This was followed by development of Mobitz type II heart block shortly after induction. Mobitz type II is a type of second-degree AV block and is due to disease of the distal conduction system characterized on the ECG by intermittently nonconducted P waves. The medical significance of this is that it can progress to complete heart block and cardiac arrest. Mobitz type II heart block is irreversible and not secondary to autonomic tone or nodal blocking agents like Mobitz type I heart block. It occurs more commonly in patients with structural heart disease and is often associated with myocardial ischemia or fibrosis of the myocardium. The definitive treatment for this condition is an implanted pacemaker [8].

The split P waves present on our patient’s preoperative ECG suggest that he had atrial conduction abnormalities prior to surgery and was at risk for further arrhythmias. We cannot definitely rule out other causes for his bradycardia, split P waves, and subsequent Mobitz type II arrhythmia, such as a “high” spinal or left-bundle branch block, but it is likely that the patient had some sort of ischemic and/or infiltrative disease, possibly related to his previous right coronary artery disease, and those conduction abnormalities were related. Whatever the underlying cause, anesthesiologists should be aware that the presence of widely split P waves on an ECG indicates abnormal atrial conduction that potentially may proceed to more serious cardiac arrhythmias during any anesthetic procedure.

## Data Availability

Not applicable.

## Abbreviations

HIPAA: Health Insurance Portability and Accountability Act
ECG: Electrocardiogram
BPM: Beats per minute
MAP: Mean arterial pressure
